# Inhibition of proliferation of rat lens epithelial cell by overexpession of *KLF6*

**Published:** 2011-04-27

**Authors:** Ying Su, Feng Wang, Dan Zhou, Weiqi Gao, Qi Hu, Hao Cui, Jie Zhang

**Affiliations:** 1 Department of Ophthalmology, First Clinic College of Harbin Medical University, Harbin, China; 2 Department of Ophthalmology, University of Nebraska Medical Center, Omaha, NE; 3 Redox Biology Center, University of Nebraska-Lincoln, Lincoln, NE

## Abstract

**Purpose:**

Proliferation of lens epithelial cell (LEC) is main principle for posterior capsular opacity (PCO) following surgery. We investigated whether overexpression of Krüppel-like factor 6 (*KLF6*) can be employed to increase protein 21 (p21) and protein 27 kinase inhibition protein 1 (p27 ^kip1^) levels and its effect on proliferation of LEC.

**Methods:**

A plasmid containing *KLF6* cDNA was used to increase the level of KLF6 protein in rat lens epithelial cells (rLEC) which can lead to consequent degradation of p21 and p27 ^kip1^. Cell proliferation was assayed by cell counts and bromodeoxyuridine (BrdU) Incorporation.

**Results:**

western blot analysis showed increased levels of KLF6, p21, and p27^kip1^ in cells transfected with pKLF6 cDNA. pKLF6 cDNA transfected cells showed less compared with control cells in vitro.

**Conclusions:**

pKLF6 cDNA inhibited cell proliferation and decreased cell viability of LEC by unregulation of p21 and p27 ^kip1^.

## Introduction

Posterior capsular opacification (PCO), which is also termed secondary cataract, is a common long-term complication of modern cataract surgery [1]. At present, the only effective treatment of PCO is Nd:YAG laser capsulotomy, which involves clearing the visual axis by creating a central opening in the opacified posterior capsule [2,3].

The tumor suppressor Krüppel-like factor 6 (KLF6) is a member of the Kruppel-like zinc finger transcription factor family of proteins, which are involved in regulating differentiation, development, cellular proliferation, growth-related signal transduction, and apoptosis.

KLFs are a subclass of the zinc finger family of DNA-binding transcription factors. *KLF6* (also known as core promoter element binding protein [COPEB] or GC-rich sites binding factor [GBF]) was independently cloned from liver, placenta, and leukocyte cDNA libraries by several groups [4-7]. *KLF6* was initially shown to be rapidly induced in activated hepatic stellate cells, the key fibrogenic cell type in liver injury and repair, implicating this factor as playing a role in tissue injury.

In present study, we examined if transfection with *KLF6* cDNA could upregulate protein 21 (p21) and protein 27 kinase inhibition protein 1 (p27^kip1^) in rat LEC (rLEC) and their effect on rLEC proliferation in vitro.

## Methods

### Reagents

KLF6, p21, and p27 kip1 antibody were purchased from Santa Cruz biotechnology Inc., (Santa Cruz, CA). Dulbecco’s modified Eagle’s medium medium (DMEM), streptomycin, and penicillin were purchased from Gibco (Carlsbad, CA). Fetal calf serum was purchased from Invitrogen (Carlsbad, CA). Hanks solution was purchased from Hyclone (Logan, UT). Poly-lysine was purchased from Sigma (St. louis, MO). Culture plates were purchased from BD Biosciences (San Jose, CA). Hybond-P polyvinylidene difluoride (PVDF) membrane was purchased from Amersham Pharmacia Biotech (Piscataway, NJ). Methylthiazolyltetrazolium (MTT) was purchased from Sigma. MTT cell proliferation kits were purchased from ATCC (Manassas, VA). The Cell Counter was purchased from Coulter (Z1; Jacksonville, FL).

### Cell culture of rLEC

All experimental procedures were in accordance with our institutional guidelines for animal care and the Guide for Care and Use of Laboratory Animals published by the US National Institutes of Health (NIH publication no. 85–23, revised 1996).

The Wistar rats (Harbin Huakang company, Harbin, Heilongjiang province, China) used in this investigation were handled in according to the tenets of the ARVO Statement for the Use of Animals in Ophthalmic and Vision Research. Six-week-old rats were killed by CO_2_ inhalation. The entire eye was removed and dipped in 75% ethanol for 30 s, and then it was opened from the posterior segment. The dissection procedure was performed with sterile instruments under a laminar flow hood. Lenses were carefully dissected by a posterior approach and washed three times in phosphate-buffered saline (PBS) to remove attached pigments and vitreous. The capsule epithelium was dissected by fine forceps and placed in a 35 mm^2^ culture dish where it adhered to the plastic. Some drops of culture medium were applied to the epithelium specimens to prevent drying, and the dishes were put in a humidified CO_2_ incubator (5% CO_2_, 37 °C) for 6 h to allow firm attachment of the capsules. Another 2 ml of culture medium was then added, and the capsules were left for 3 or 4 days before the first trypsinization and subculturing. The medium, which was changed every 2 days, was DMEM with 20% fetal bovine serum (FBS), supplemented with 2 mM L-glutamine, 100 U/ml penicillin and 100 μg/ml streptomycin.

rLECs were cultured in a six-well plate with a sterile coverslip. All culture media were modified Eagle's medium with 10% fetal bovine serum (FBS), supplemented with 2 mM L-glutamine, 100 U/ml penicillin and 100 μg/ml streptomycin. Cells used in subsequent experiments were generally from passages two to three. All cells were grown at 37 °C with 5% CO_2_ ventilation [8].

### *KLF6* overexpression plasmid construct

To enforce *KLF6* expression in the cells, the full-length cDNA (852 bp) of *KLF6* containing NheI and XhoI restriction digestive sites at both ends was amplified using a PfuUltra Hotstart DNA Polymerase Kit (Biocompare company, Palo Alto, CA). The amplified *KLF6* DNA fragment was subsequently cloned into a pTRE vector (Takara Bio Inc, Otsu, Shiga) [9].

rLEC transfected with plasmid containing *KLF6* cDNA, empty vector only, and medium served as the experimental group, vehicle control group, and blank control group, respectively. Transfection was performed in 60 mm plates using 3µg (1 µg/µl) vector in 10 µl of Metafectene Pro reagent (Biontex, Martinstried, Germany). After 48 h of transfection, cells were treated with G418 (Life Technologies, Carlsbad, CA) for 2 weeks for positive clone selection. After G418 treatment, several stable transfectant cells were cloned. Each clone was screened for expression of KLF6 by western blot analysis.

### Western blot analysis

rLEC were separately by ultrasonic disruption in a lysis buffer. Protein Protein concentration was measured using absorbance spectroscopy. Protein was separated on a 10% SDS-polyacrylamide gel and transferred to a PVDF membrane (Biocompare company, Palo Alto, CA). After blocking with 5% nonfat milk, the membranes were incubated with primary antibodies against KLF6, p21, and p27 ^kip1^ (Santa Cruz biotechnology Inc., Santa Cruz, CA) overnight at 4 °C, followed by incubation with secondary antibodies (Santa Cruz biotechnology Inc.). Densitometry of the bands was performed with quantity one analysis software.

### Cell proliferation and bromodeoxyuridine (BrdU) incorporation

For bromodeoxyuridine (BrdU) incorporation, rLEC cells growing on coverslips were fixed with 4% paraformaldehyde in vitro for 30 min at 4 °C and washed in 0.1M PBS (pH 7.4) with 1% Triton. The cells were then incubated in HCl (1 N) for 10 min on ice followed by HCl (2 N) for 10 min at room temperature, followed by incubating overnight with anti-BrdU or a combination of anti-BrdU antibody and then treated with secondary antibodies to visualize the anti-BrdU labeled cells. The nuclei were simultaneously stained with 10 µg/ml of 4V, 6-diamidino-2-phenylindole. Cells with different BrdU-incorporation patterns were analyzed and counted with a traditional light microscope (CX21, Olympus Inc, Tokyo, Japan) [8].

### Statistical analysis

The data were analyzed by the two-tailed Student *t*-test using SPSS (IBM Inc, Beijing, China) 10.0 and p<0.05 was considered significance.

## Results

### Upregulation of KLF6 protein by pKLF6 cDNA in rLEC

**Figure 1:**
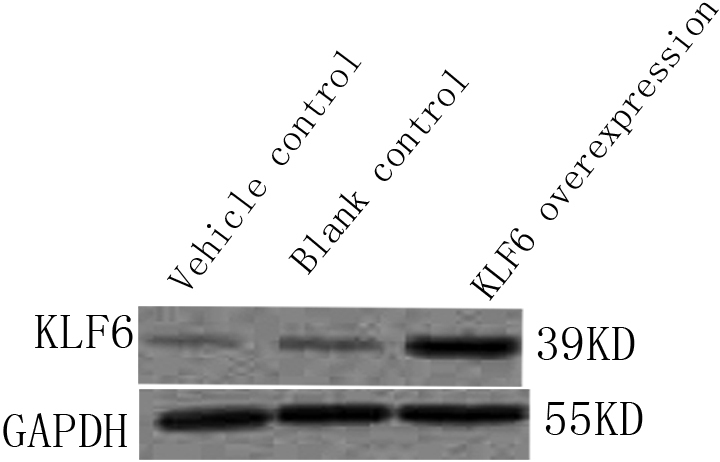
Upregulation of KLF6 protein by pKLF6 cDNA in rLEC. KLF6 expression can be detected in rLEC’s compared to the vehicle control group and blank control group (lane 1 and 2) in vitro. However, there was increased expression of KLF6 in the experimental group, which indicated that transfection with pKLF6 cDNA can increase expression of KLF6 in rLEC (lane 3) in vitro. glyceraldehyde-3-phosphate dehydrogenase (GAPDH) served as a loading control.

KLF6 expression can be detected in rLEC’s compared to the vehicle control group and blank control group (Figure 1, lane 1 and 2) in vitro. However, there was increased expression of KLF6 in the experimental group, which indicated that transfection with pKLF6 cDNA can increase expression of KLF6 in rLEC (Figure 1, lane 3) in vitro.

### Upregulation of p21 in rSF transfected with pKLF6 cDNA

**Figure 2:**
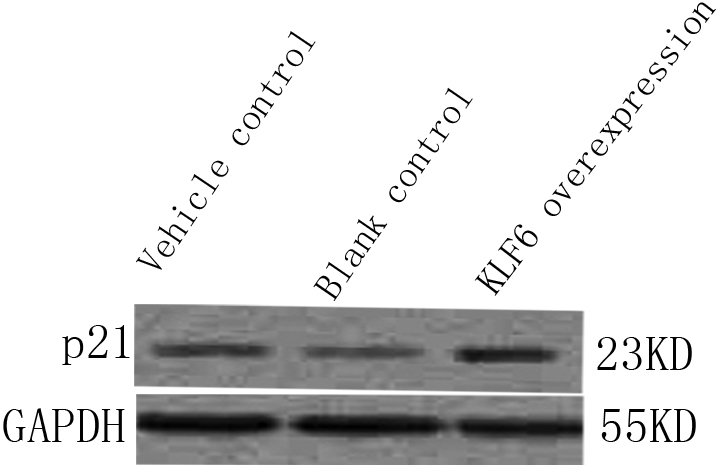
Upregulation of p21 in rSF transfected with pKLF6 cDNA. Little expression of p21 protein in the rLEC transfected with vehicle (lane 1) or without transfection (lane 2). Upregulation of p21 was detected in rLEC transfected with pKLF6 cDNA (lane 3). GAPDH served as loading control.

Little expression of p21 protein in the rLEC transfected with vehicle (Figure 2, lane 1) or without transfection (Figure 2, lane 2). Upregulation of p21 was detected in rLEC transfected with pKLF6 cDNA (Figure 2, lane 3).

### Upregulation of p27^kip1^ in rSF transfected with pKLF6 cDNA

**Figure 3:**
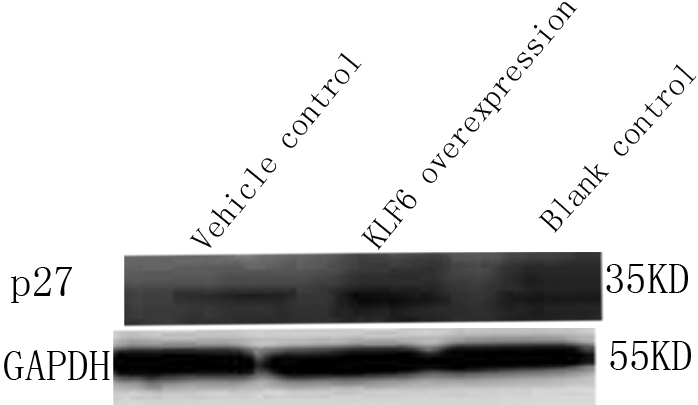
Upregulation of p27^kip1^ in rSF transfected with pKLF6 cDNA. Expression of p27^kip1^ increased in rLEC transfected with pKLF6 cDNA (lane 2) compared with the blank control (lane 3) or vehicle control (lane 1). GAPDH served as loading control.

Expression of p27^kip1^ increased in rLEC transfected with pKLF6 cDNA (Figure 3, lane 2) compared with the blank control (Figure 3, lane 3) and vehicle control (Figure 3, lane 1).

### Incorparation of Brdu

**Figure 4:**
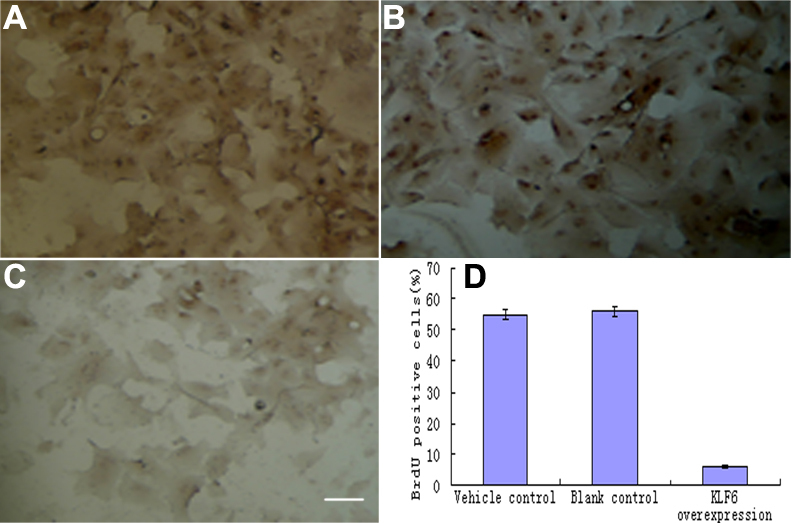
Cell proliferation assay by Brdu. Expression of BrdU decreased in pKLF6 cDNA transfectant cells (**C**) compared with rLEC of the vehicle control (**A**) and blank control (**B**). Cell counting showed that Brdu positive cells in rLEC transfected with pKLF6 cDNA significantly decreased compared with control cells (p<0.01 versus vehicle and blank control, **D**).

Expression of BrdU decreased in pKLF6 cDNA transfectant cells (Figure 4C). compared with rLEC of vehicle control (Figure 4A) and blank control (Figure 4 B) after cell counting showed that Brdu positive cells in rLEC transfected with pKLF6 cDNA significantly decreased compared with control cells (p<0.01 versus vehicle and blank control; Figure 4D).

## Discussion

Lens epithelial cells (LECs) left behind in the capsular bag after any type of extracapsular cataract surgery are mainly responsible for PCO development [10]. Proliferation, migration, epithelial-to-mesenchymal transition (EMT), collagen deposition, and lens fiber regeneration of LECs are the main causes of opacification

Fibrosis-type PCO is caused by the proliferation and migration of LECs, which undergo EMT, resulting in fibrous metaplasia and leading to significant visual loss by producing folds and wrinkles in the posterior capsule [11]. Pearl-type PCO is caused by the LECs located at the equatorial lens region (lens bow) causing regeneration of crystallin-expressing lenticular fibers and forming Elschnig pearls and Soemmering ring, responsible for most cases of PCO-related visual loss [12].

There are several urgent reasons to eradicate PCO. First, PCO remains the most common complication of cataract surgery [13,14]. Posterior capsular opacification is even more threatening in young adults and children, with a higher incidence, quicker onset, and greater amblyogenic effect. Migration of human LECs plays an important role in the remodeling of the lens capsule [15,16] and is associated with matrix metalloproteinase activity in the lens [17]. Matrix metalloproteinases are a group of proteolytic enzymes, essential for cell migration and cell-mediated contraction after wound healing [18].

KLF6 (Kruppel-like factor 6) is a tumor suppressor protein that is down-regulated or mutated in several types of cancers, including prostate cancer [19-21]. KLF6 is a zinc finger transcription factor that binds to a GC box and regulates the expression of target genes. It has been shown that KLF6 suppresses tumor growth through activating protein 21 wild-type activated fragment 1/cyclin inhibitor protein 1 (p21 WAF1/Cip1), an inhibitor of the cyclin-dependent kinases, in both cultured cells and a transgenic mouse model [22]. KLF6 also directly interacts with cyclin D1 to suppress cyclin-dependent kinase 4 and causes cell cycle arrest [23].

Of particular note with respect to potential therapeutic targeting are our studies demonstrating not only that overexpression of KLF6-SV1 is associated with decreased survival and metastatic spread of certain cancers but also that siRNA-mediated inhibition of KLF6-SV1 has such dramatic effects on tumor behavior in vitro and in vivo [24-26].

Our previous result indicated that s-phase kinase protein 2 (*skp2*) siRNA can inhibit proliferation of proliferation of rLEC and cell viability decreased from the 6th day to the 12th day after transfection. *Skp2* siRNA transfection also increased expression of p27^kip1^. p27^kip1^ is important for regulation of cell cycle. Our present data showed that tansfection with pKLF6 cDNA prevented rLECs proliferation in vitro especially from the 10th day after transfection. Thus, it might be used to prevent PCO after cataract surgery.

In summary, this study has delineated the role of KLF6 in inhibition the proliferation of rLECin vitro. Increased expression of p21 and p27^kip1^ after transfection with *KLF6* cDNA suggest their important role for inhibition of cell proliferation. Our result may be the foundation for further research on relation between KLF6 SV1 and proliferation of LEC.
